# High-Dose Dependence and Cognitive Side Effects to Medical Prescription of Etizolam

**DOI:** 10.3389/fpsyt.2020.601827

**Published:** 2020-11-04

**Authors:** Stefano Tamburin, Elisa Mantovani, Anna Bertoldi, Angela Federico, Rebecca Casari, Fabio Lugoboni

**Affiliations:** ^1^Department of Neurosciences, Biomedicine and Movement Sciences, University of Verona, Verona, Italy; ^2^Addiction Medicine Unit, Department of Medicine, Verona University Hospital, Verona, Italy

**Keywords:** benzodiazepine, BZD, cognition, dependence, etizolam, substance use disorder (SUD), neuropsychology

## Abstract

**Introduction:** The use of novel designer drugs has increased worldwide over the years. Etizolam is a designer benzodiazepine (BZD) that has raised concern because of its growing non-medical use, liability to tolerance and dependence, and related harms. Studies exploring the abuse liability and cognitive effects of etizolam outside the therapeutic doses are lacking.

**Aims:** To explore the abuse liability of etizolam and the characteristics of patients affected by etizolam high-dose dependence in a nationwide tertiary referral addiction unit. To document the cognitive changes to etizolam high-dose use.

**Design and Methods:** Sociodemographic and clinical data on subjects with etizolam high-dose use were retrospectively collected from a database of 1,293 patients consecutively admitted to the Addiction Medicine Unit, Verona University Hospital, Italy for detoxification from high-dose BZDs or Z-drugs dependence. Thorough neuropsychological testing explored the cognitive side effects of high-dose etizolam use.

**Results:** We found eleven etizolam high-dose users, of which eight used etizolam only, and three used etizolam with other BZDs/zolpidem. All the patients were prescribed etizolam for medical reasons, i.e., anxiety and/or insomnia. Neuropsychological evaluation showed deficits of working memory, visuospatial memory and executive function in a 27-year-old woman who used etizolam 15 mg daily.

**Discussion:** Our findings suggest that abuse and dependence liability of etizolam should be considered a public health and social problem. They offer preliminary evidence on the cognitive side effects of etizolam high-dose use.

**Conclusions:** This report offers new information on the potential harms of etizolam in patients who are prescribed this drug for medical reasons.

## Introduction

Benzodiazepines (BDZs) are gamma-amino-butyric acid type A (GABA-A) receptor positive allosteric modulators widely prescribed for anxiety, insomnia and other conditions (1).

The increasing use of novel designer BZD derivatives has been recently reported in countries, where these chemical compounds do not have marketing authorization as medicinal products (2).

Etizolam is a short-acting (half-life 5–7 h) thienodiazepine designer BZD with high affinity for the GABA-A receptor and anxiolytic and sedative properties (3). Etizolam is currently approved for therapeutic use and marketed in three countries, namely India, Italy and Japan, but available from the Internet for research purposes worldwide (4). The few available comparative studies reported that etizolam may induce less tolerance than lorazepam and may have lesser sedative effects than alprazolam and diazepam (4). The lower allosteric potency at the α1 subunit of the GABA-A receptor has been proposed as one reason for the reduced liability of etizolam to tolerance and dependence (5).

A consistent increase in the non-medical use and the illicit drug market of etizolam has been reported since 2014, being this drug implicated in several deaths in Scotland, United Kingdom, and to a lesser extent in the United States and Sweden (4, 6). A recent review concluded that few harms are documented with the therapeutic use of etizolam, being predominantly related to its non-medical use in illicitly manufactured pills and in the context of mixed-drug toxicity, in particular in combination with opioids (7). The World Health Organization Expert Committee on Drug Dependence (WHO-ECDD) considered etizolam abuse or dependence liability as an effectively public health and social problem (4). Some questions on etizolam side-effects profile are still unanswered. Evidence on etizolam safety is based on preclinical studies and case reports. Common adverse effects of etizolam include drowsiness, sedation and slurred speech, but this drug is considered generally well-tolerated in terms of cognitive side effects (4). The auditory P300 was found to be prolonged with etizolam, but this slowed brain response showed habituation, while attention and memory appeared to be unaffected by etizolam (8). Therapeutic etizolam doses (0.25–1 mg) had no effect on cognition in patients with anxiety (9) and on psychomotor performance and vigilance (10). Cognitive effects to higher doses of etizolam are still unexplored.

High-dose dependence of BZDs or related Z-drugs (e.g., zolpidem, zopiclone, eszopiclone, zaleplon), i.e., daily intake ≥5 times the recommended maximum daily dosage (1) is an emerging substance use disorder estimated to affect 0.16% of the adult population in Switzerland (11), associated to poor quality of life (12), and cognitive dysfunction (13). Data on etizolam high-dose dependence are lacking.

This report is aimed to (a) explore the liability of etizolam to abuse and the characteristics of patients affected by etizolam high-dose dependence in a nationwide tertiary referral addiction unit; (b) document the cognitive changes to etizolam in a high-dose user who underwent thorough neuropsychological evaluation.

## Methods

Subjects with high-dose etizolam were retrospectively collected from a database of 1,293 patients (650 men, 643 women) aged >18 years and admitted (January 2003–December 2019) to the Addiction Medicine Unit, Verona University Hospital, Italy, a nationwide tertiary referral center for detoxification from high-dose BZD/Z-drug dependence with slow flumazenil infusion (14).

High-dose BZD/Z-drug dependence was defined according to DSM-IV-TR criteria (15) with use lasting >6 months, daily dosage exceeding at least 5 times the recommended maximum intake (i.e., >50 mg of daily diazepam dose equivalent, DDDE), otherwise problematic use of BZD/Z-drug, such as mixing different molecules, escalating dosage, obtaining them by illegal means, and using them to enhance the effect of other substances (14).

We collected socio-demographic and clinical variables of the patients. The dosage of BZD/Z-drugs was based on self-report. DDDE (mg) was calculated according to conversion tables (14). The diagnosis of psychiatric disorders was based on screening tests, diagnostic interviews, and previous psychiatric assessments or evaluations, when available.

Neuropsychological evaluation explored verbal memory, working memory, visuospatial memory, attention and executive function. Verbal memory was assessed by means of the Italian versions of the Verbal Paired Association (VPA) (16) and the Digit Span Forward Test (DSFT) (17). Working memory was evaluated with the Digit Span Backward Test (DSBT) (17) and Paced Auditory Serial Addition Test (PASAT-3) (18). Visuospatial memory was explored with the 10/36 Spatial Recall Test (SPART) (18). Attention was measured with the Trail Making Test Part A (TMT-A) and the Symbol Digit Modalities Test (SDMT) (18). Executive function was evaluated by means of Trail Making Test Part B (TMT-B) (18) and the Stroop test (19). Results were standardized as Z-scores. Cognitive testing was performed 1 month before detoxification treatment.

All patients underwent a detoxification protocol with slow subcutaneous flumazenil infusion (40.5 μg/h for 24 h/day for 7 days) with a prophylactic antiepileptic treatment (14).

The study was conducted according to the Declaration of Helsinki and approved by the ethics committee of the Verona University Hospital. All the patients gave written informed consent to the study.

## Results

Among the patients admitted from January 2003 to December 2019, we found 11 patients (4 men, 7 women) who used high-dose etizolam either as the only BZD (8 patients) or with other BZDs or Z-drugs (3 patients; bromazepam: 1; lorazepam and zolpidem: 1, clonazepam and triazolam: 1). All the patients were prescribed etizolam for medical use (anxiety: 5 patients; sleep disorders: 1; both reasons: 5) and obtained the drug through a prescription and a pharmacy. The number of patients was stable across years (2003–2007: 2 patients; 2008–2011: 3; 2012–2015: 4; 2016–2019: 2). Sociodemographic and clinical features of patients are reported in Table 1.

**Table 1:** Sociodemographic and clinical characteristics of the patients.

**Characteristics**	
Sociodemographic variables	
Gender (men/women)	4/7
Age^a^	41.4 ± 7.7; 41; 27–52
Education (grade school/high school/university)	4/3/4
Employment (unemployed/employed)	3/8
Marital status (single/engaged or married)	6/5
Clinical variables	
Etizolam daily dosage	27.3 ± 29.3; 15; 5–100
Etizolam formulation (tablet/drops/both)	3/7/1
Concomitant abuse of other BZD/Z-drugs (yes/no)	3/8
DDDE (mg)^a^	272.7 ± 263.5; 150; 70–1,000
BZD/Z-drug use duration (mos)^a^	36.6 ± 26.0; 24; 10–84
Age of first BZD/Z-drug intake^a^	24.6 ± 8.3; 21; 14–37
Reason for prescription (anxiety/sleep disorders/both)	5/1/5
Poly-drug use (yes/no)	7/4
Alcohol (yes/no)	5/6
Opioids (yes/no)	1/10
Cocaine (yes/no)	2/9
Cannabinoids (yes/no)	2/9
Tobacco	5/6
Psychiatric disorders (yes/no)	9/2
Anxiety disorders (yes/no)	6/5
Major depression (yes/no)	4/7
Other psychoses (yes/no)	1/10
Personality disorders (yes/no)	2/9

a *Mean ± SD; median; range. BZD, benzodiazepine; DDDE, daily diazepam dose equivalent (sum of DDDEs for all BZDs and Z drugs in case of concomitant abuse of other BZD/Z-drugs); Mos, months*.

The remaining 1,282 patients used other BZD/Z-drugs high-doses [for further details see (12, 13)].

**Figure 1:**
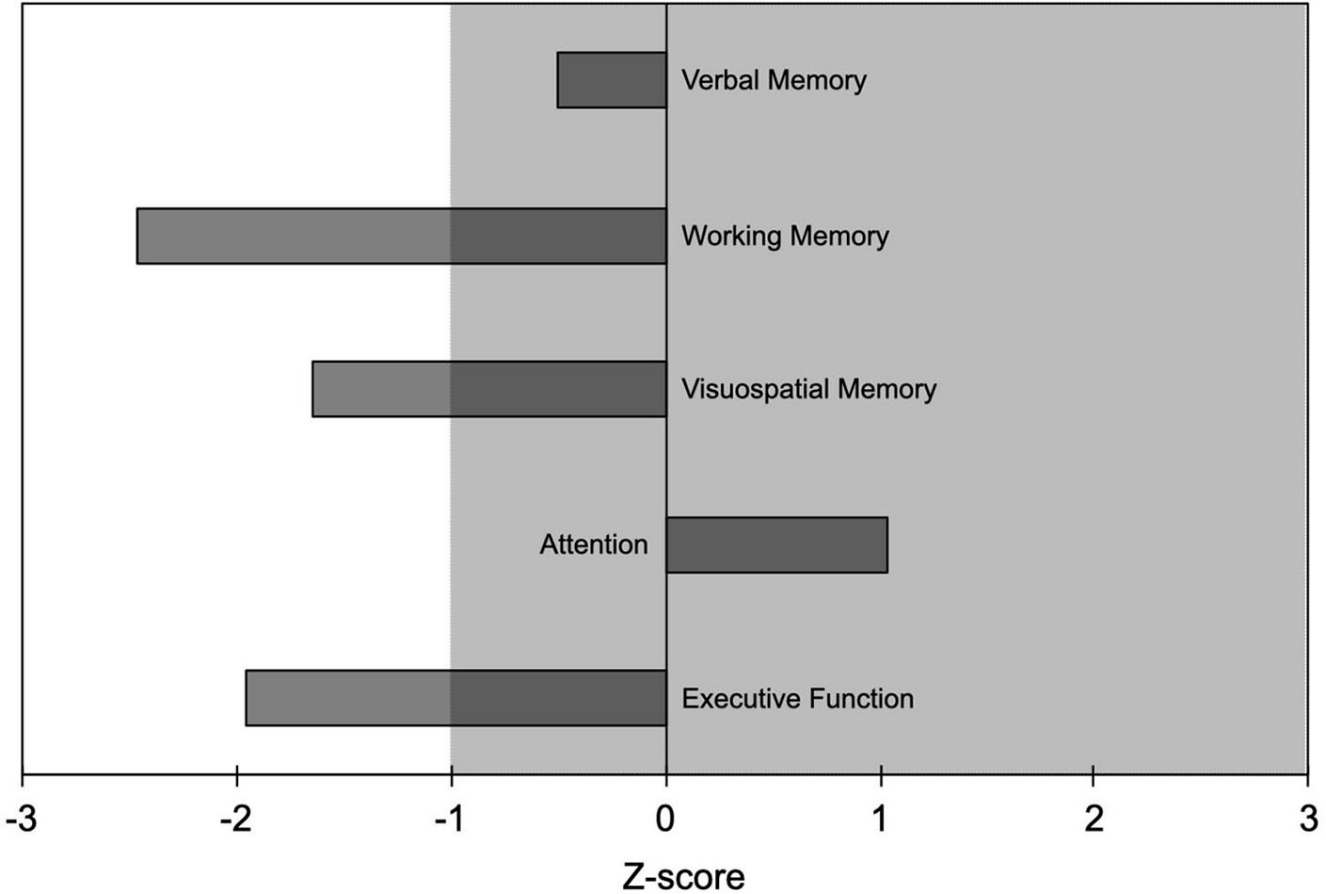
Results of the neuropsychological evaluation from one patient (woman, age 27 years, education 8 years) who took etizolam 15 mg daily. Neuropsychological findings are reported as Z-scores with negative values indicating worse performance and positive values indicating better performance than the average value of the normal population. Abnormal values are worse than mean−1 SD; i.e., Z-scores <-1 indicate abnormal values. Shaded area indicates normal range of values.

One patient (woman, 27 years, education 8 years; 15 mg of etizolam daily) underwent neuropsychological evaluation that showed working memory, visuospatial memory, and executive function to be outside normal values (i.e., >1 SD worse than normal controls; Figure 1). No medical conditions or other substance use disorder that could have contributed to the cognitive deficits were reported.

## Discussion

To the best of our knowledge, this is the first report of etizolam high-dose dependence in 11 patients who received etizolam for medical reasons (anxiety and/or sleep disorders). Our patients received on average 27.3 ± 29.3 mg etizolam daily, which is nearly ten times the maximum recommended daily dosage (i.e., 3 mg), with one patient taking 100 mg daily, i.e., >30 times the maximum daily dosage. These findings support the conclusion of the recent WHO-ECDD report that abuse/dependence liability of etizolam should be considered as a public health and social problem (4). They are also in keeping with individual users reports on forums like Bluelight.org (20) and Erowid.org (21) that describe tolerance, craving and withdrawal to etizolam (7).

Etizolam high-dose users represented 0.9% of our whole sample and did not increase during the nearly 20 years covered by our large database, suggesting some concern, but stable figures across time. The big size difference between etizolam and other BZDs/Z-drugs high-dose users hampered a reasonable statistical comparison. However, when compared to high-dose users of lormetazepam, i.e., the most common BZD of high-dose use in Italy (22), etizolam high-dose users appear to be more frequently women, younger, more frequently employed, with lower DDDE, shorter BZD/Z-drug use duration, smaller age of first BZD/Z-drug intake and more frequent poly-drug use. These findings suggest that some populations of patients might be more prone to non-medical use and dependence of etizolam. In particular, they confirm the risk of etizolam harms in patients with other substance use disorders (4).

The small number of etizolam high-dose users in our large sample is likely related to the number of prescriptions in the general population. Etizolam does not stand among the ten most prescribed BZD active principles in Italy, in that its defined daily dose (DDD) in 2018 was <0.5/1,000, while that of the most commonly ones ranged from 13.2 for lormetazepam to 0.7 for flurazepam (23). The absence of data on etizolam prescription in Italian population, however, hampers the estimation of the conversion-rate from etizolam prescription to addiction.

Etizolam negatively influenced most of the cognitive domains in the patient who underwent neuropsychological testing, in particular working memory, visuospatial memory, and executive function, some of them being <2 SDs worse than normal values. This finding, despite being preliminary since stemming from a single patient, extends the notion that high doses of BZDs have an impact on cognition, even in younger patients (13, 24). BZDs cognitive side effects have been suggested to be related to the function of the GABA-A receptor α1 (responsible for anterograde amnesia) and the α5 subunits, which are involved in cognition, learning and memory (25). Based on animal studies showing etizolam lower affinity for the GABA-A receptor α1 subunit than the α5 one (5), we speculate that the cognitive effects of etizolam high-dose intake in our patient might be mainly mediated by the interaction with the α5 subunit.

The strengths of this study are that it represents the first series of etizolam high-dose users, and offers new information on the harms of this BZD derivative from a nationwide referral center in one of the few countries where it is marketed. The limitations are the retrospective design, the absence of systematic quantitative BZD measures to verify self-reported data, and neuropsychological data from a single patient that suggest caution in generalizing our findings on cognitive side effects of etizolam.

In conclusion, a small number of patients who use etizolam for therapeutic reasons appear to transition to high-dose use requiring specialist care. This report offers new information on the potential harms related to etizolam and extends them to patients who are prescribed this drug for medical reasons. Future studies should confirm our findings in larger populations and in other countries where etizolam is marketed.

## Data Availability Statement

The raw data supporting the conclusions of this article will be made available by the authors, without undue reservation.

## Ethics Statement

The studies involving human participants were reviewed and approved by Ethics Committee of the Verona University Hospital. The patients/participants provided their written informed consent to participate in this study.

## Author Contributions

ST, EM, AB, AF, RC, and FL designed the study and gathered the data. ST analyzed the data. ST, EM, AB, AF, and RC drafted the manuscript. FL and ST revised the manuscript. All authors approved the final version of the manuscript.

## Conflict of Interest

The authors declare that the research was conducted in the absence of any commercial or financial relationships that could be construed as a potential conflict of interest. The reviewer, AM, declared a past co-authorship with one of the authors, FL, to the handling Editor.
